# Tamoxifen improves cytopathic effect of oncolytic adenovirus in primary glioblastoma cells mediated through autophagy

**DOI:** 10.18632/oncotarget.2897

**Published:** 2015-03-02

**Authors:** Ilya V. Ulasov, Nameeta Shah, Natalya V. Kaverina, Hwahyang Lee, Biaoyang Lin, Andre Lieber, Zaira G. Kadagidze, Jae-Guen Yoon, Brett Schroeder, Parvinder Hothi, Dhimankrishna Ghosh, Anatoly Y. Baryshnikov, Charles S. Cobbs

**Affiliations:** ^1^ Swedish Neuroscience Institute, Seattle, WA, 98122, USA; ^2^ NN. Blokhin Cancer Research Center, RAMN, Moscow, Russia, 115478; ^3^ University of Washington, Seattle, WA, 98122, USA; ^4^ Institute of Experimental Diagnostic and Biotherapy, NN. Blokhin Cancer Research Center, RAMN, Moscow, Russia, 115478; ^5^ Current address: Division of Nephrology, University of Washington, Seattle, 98109, USA

**Keywords:** brain tumor, adenovirus, survivin, CRAd, autophagy

## Abstract

Oncolytic gene therapy using viral vectors may provide an attractive therapeutic option for malignant gliomas. These viral vectors are designed in a way to selectively target tumor cells and spare healthy cells. To determine the translational impact, it is imperative to assess the factors that interfere with the anti-glioma effects of the oncolytic adenoviral vectors. In the current study, we evaluated the efficacy of survivin-driven oncolytic adenoviruses pseudotyping with adenoviral fiber knob belonging to the adenoviral serotype 3, 11 and 35 in their ability to kill glioblastoma (GBM) cells selectively without affecting normal cells. Our results indicate that all recombinant vectors used in the study can effectively target GBM *in vitro* with high specificity, especially the 3 knob-modified vector. Using intracranial U87 and U251 GBM xenograft models we have also demonstrated that treatment with Conditionally Replicative Adenovirus (CRAd-S-5/3) vectors can effectively regress tumor. However, in several patient-derived GBM cell lines, cells exhibited resistance to the CRAd infection as evident from the diminishing effects of autophagy. To improve therapeutic response, tumor cells were pretreated with tamoxifen. Our preliminary data suggest that tamoxifen sensitizes glioblastoma cells towards oncolytic treatment with CRAd-S-5/3, which may prove useful for GBM in future experimental therapy.

## INTRODUCTION

Glioblastoma multiforme (GBM) are malignant brain tumors in adults and children with no known cure [1]. Advances in molecular biology of GBM reveal new opportunities for gene therapy using adenoviral vectors. Recent preclinical studies have shed light on the utility of adenovirus as a vector for anti-GBM therapy [2–5]. These adenoviruses represent an attractive mode of delivering therapeutic load as these viruses exhibit high transduction efficiency for cancer cells and spread effectively within the tumor tissues. Adenoviral (Ad) vectors of subgroup C have been extensively studied. Among this subgroup, adenovirus Ad5 and Ad2 have been most frequently used due to their ability to infect multiple cell types, proliferate *in vitro*, and support efficient transgene expression [6]. To date, ONYX015 and CRAd-S-pK7 vectors have emerged as potential candidates for anti-tumor therapy [7–10]. Efficiency of GBM transduction with adenoviruses is dependent on the presence of CAR (Coxsackie and adenovirus receptor, CAR), and αvβ3 or αvβ5 integrins. However, due to low level of expressions of CAR on cancer cells, transduction efficiency of adenoviruses remains restricted to those cells that have higher expression of the receptor. To circumvent the problem associated with low level of primary receptor, in the current study we have evaluated the application of adenoviral vectors pseudotyped with subgroup B type fibers as novel anti-GBM therapeutic agents.

The Ad serotypes in subgroup B represented by Ad35, Ad3 and Ad11 viruses have been associated with respiratory disease, ocular disease, and urinary tract infection [11, 12]. Group B adenoviruses have been found to be very effective in gene therapy because of their ability to infect target tissues that otherwise could poorly be transduced by commonly used adenoviral vectors. While most adenoviruses infect cells through the CAR and integrins receptors, adenoviruses in serotype group B use CD46, DSG2 and unknown X receptors [13]. Ad serotype group B viruses (e.g. Ad 5/35 virus and Ad 5/3) infect CAR-negative cell lines more efficiently than the Ad5 vector [14]. Moreover, recent studies utilizing B-subgroup adenovirus genotyped with adenovirus type 3 [15], type 11 [4, 16] and type 35 [5, 17] demonstrated significant anti-GBM effects. However, it is not clear, which of those specific modifications (fiber 3, 11 or 35) would exhibit superior transduction efficiency in primary GBM cells derived from patients. Given the fact that presence of survivin promoter significantly improves specificity of GBM transduction and CRAd induced toxicity [15], we designed a panel of survivin-driven oncolytic vectors recognizing DSG2 or CD46 receptors.

The present study was designed to evaluate GBM transduction mediated by adenoviral vectors, whose protruding fibers were engineered with fibers belonging to either adenovirus type 3, 11, or 35. Using a panel of GBM cells with different subtypes (proliferative, proneural and mesenchymal), we assessed the ability of designed vectors to infect GBM cells, and determined their anti-glioma oncolytic effect *in vitro* and *in vivo*.

## RESULTS

### DSG2, CD46 and CAR expression in brain tumor patient samples

**Figure 1:**
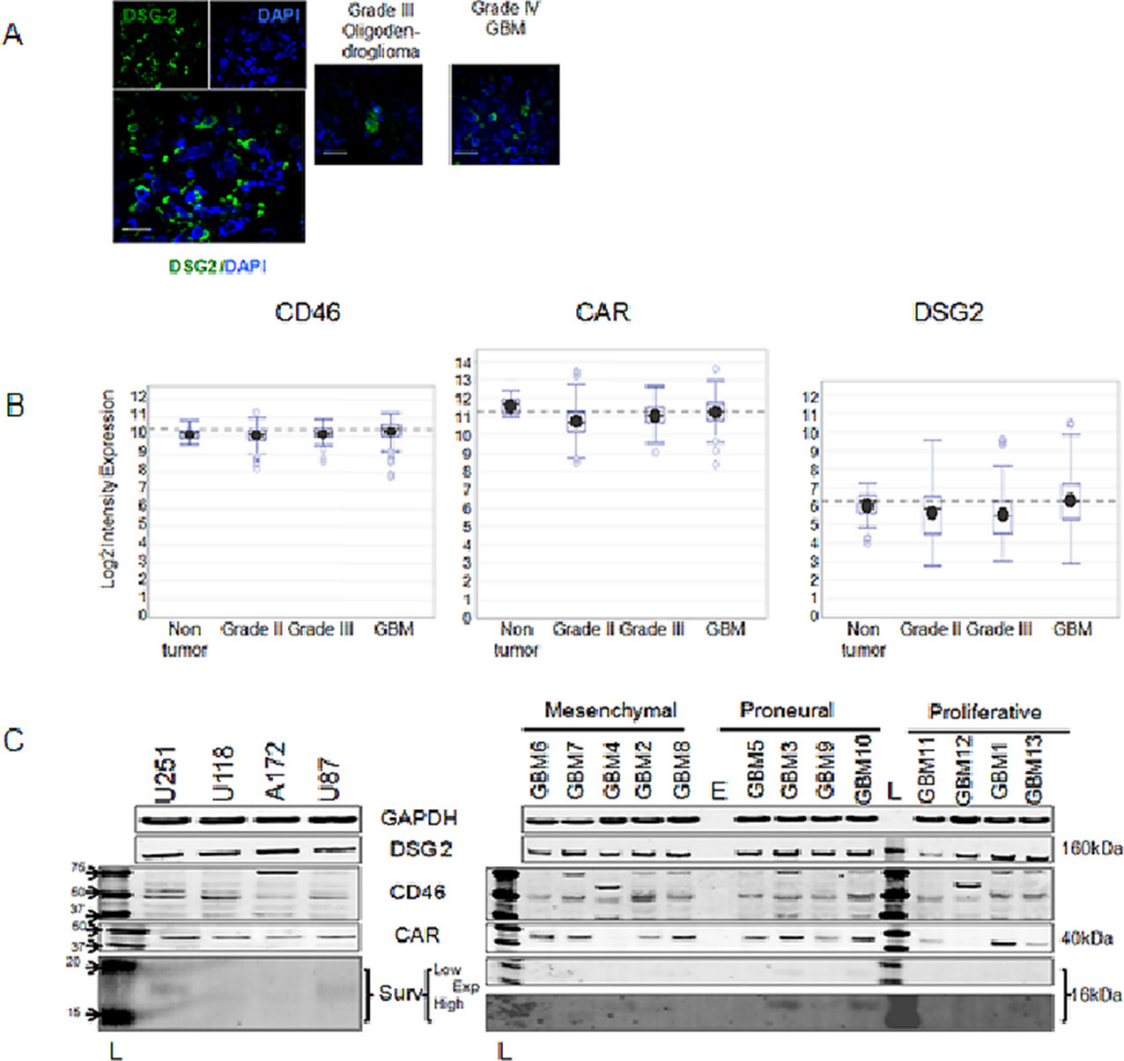
Expression of DSG2, CD46 and CAR in GBM cells **(A)** Confocal dual-immunofluorescence of DSG2 expression in brain tumor primary specimens. Left: Single and composite images of GBM tissue stained with DSG2 (green, cytoplasmic/membrane) and DAPI (blue, nuclear); Right: Composite images of brain tumor samples represent Grade III and Grade IV. Scale bars 20 microns, 600x magnification; Intensity of DSG2, CD46 and CAR expressions detected in the tumoral and non-tumoral primary samples of REMRANDT database **(B)** and presented as log2 expression. Dash line represents median value for GBM samples; **(C)** Western blotting analyses of CD46, DSG2, CD46 and survivin expressions in glioma cell lines (Left) and primary glioma cells (Right) which represent clinical settings; “E” and “L” stand for empty well and ladder. Actin was used as a loading control.

First, we analyzed whether GBM specimens express any primary adenoviral receptors, such as DSG2, CD46, or CAR on the membrane surface. To analyze DSG2 cell expression in GBM tumors, we stained primary tumors of grade III (Oligodendroglioma) and grade IV (GBM) with antibodies to DSG2 proteins. As presented (Figure 1A), primary GBM has a strong staining pattern for DSG2 compared to grade III tumors. Gene expression analysis of REMBRANDT tissue transcriptomic compendium also indicated higher expression of DSG2 and CD46 receptors relative to non-tumor regions of the brain (Figure 1B).We observed significant differences in expression of DSG2 (*p* < 0.015) and CD46 (*p* < 0.0049) in grade III relative to grade IV GBM specimens (Table 1). Additionally, DSG2 and CD46 are expressed ubiquitously in GBM tissues irrespective of GBM subtypes. To investigate whether targeting of DSG2 and CD46 receptors with adenoviral vectors would result in increased transduction, we selected primary patient-derived GBM cells of three molecular subtypes (mesenchymal, proneural and proliferative). Since cancer stem cells are believed to provide GBM recurrence [18], chemoresistance [19–21] and radio resistance [22, 23], we maintained these cells in stem cell mimicking conditions (as described in the materials and method section) to preserve stemness and characterized them for the expression of DSG2, CD46 and CAR markers. We observed no difference in DSG2 expression between 13 primary cell lines and 4 GBM cell lines. In contrast, 11 out of 13 established primary GBM cells express CAR (Figure 1C). In addition, we [24, 25] and others [26] have confirmed that human glioma cell lines: U251, A172, U118, U87 and patient-derived GBM cells strongly express CD46.

**Table 1:** Statistical significance of gene expression between samples which represent non-malignant, astrocytoma (Grade II), oligodendrodglioma (Grade III) and glioblastoma multiforme (Grade IV)

	DSG2	CXADR(CAR)	CD46
	217901_at	203917_at	207549_x_at
Non-tumor vs astrocytoma	0.721633175	7.05087E-06	0.659001322
Non-tumor vs olidendroglioma	0.597670986	0.002250223	0.66637388
Non-tumor vs GBM	3.28659E-05	0.013941438	0.002577406
Astrocytoma vs oligedendroglioma	0.762906071	0.183756462	0.310145177
Astrocytoma vs GBM	0.00022612	0.001843546	2.06494E-06
Oligodendroglioma vs GBM	0.014616933	0.204193919	0.004939011

### CRAd-S-5/3 inhibits GBM growth *in vitro* and *in vivo*

**Figure 2:**
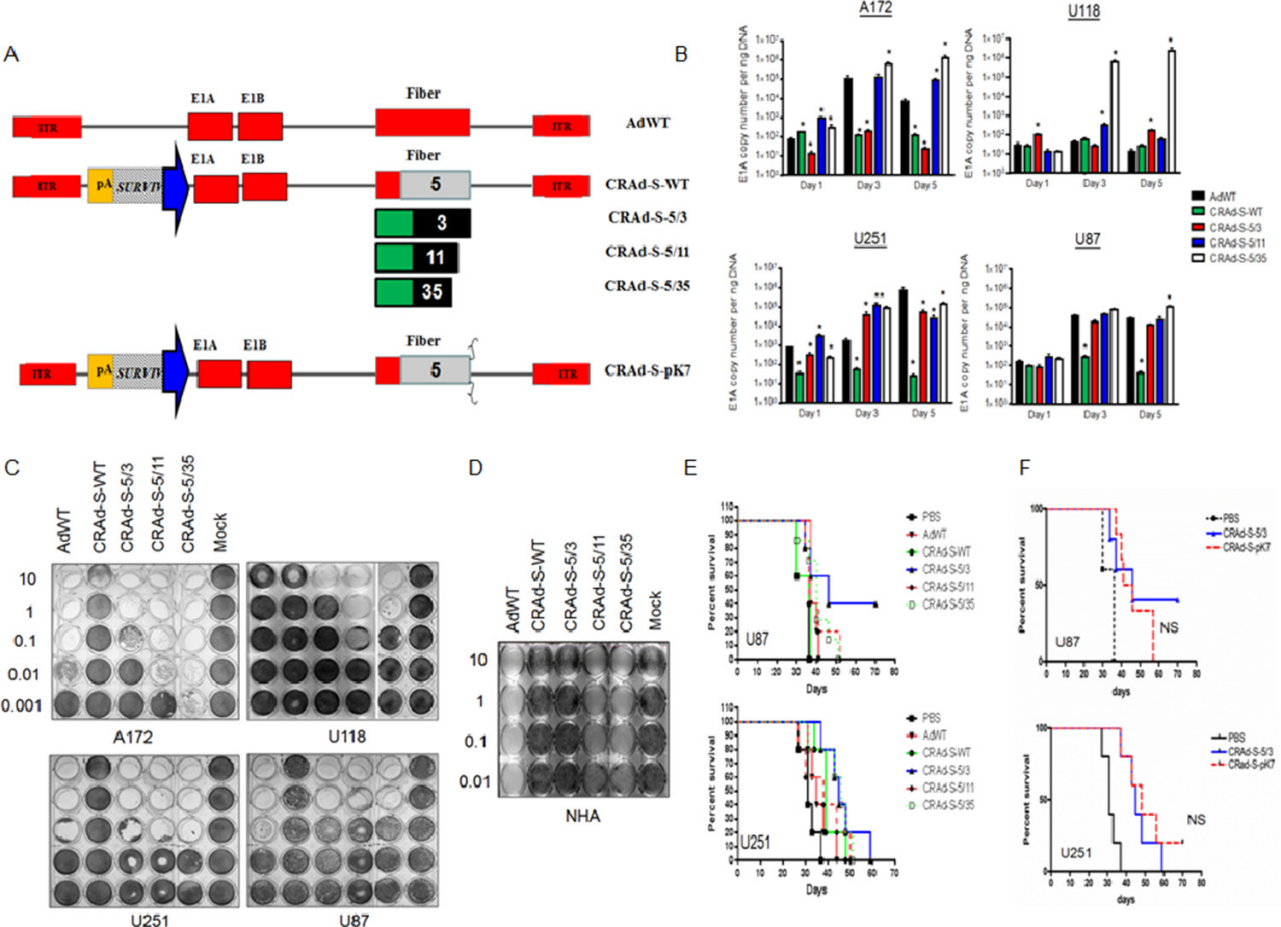
*In vitro* and *in vivo* transductional activity of oncolytic vectors using glioma cells Replication (B) and *in vitro* cytotoxic activity (C and D) of designed oncolytic vectors pseudotyped with adenoviral type B fibers. Analysis of CRAds **(A)** replication in cancer cells was conducted at the samples 1, 3 and 5 days after infection **(B)** At selective time point total DNA was isolated in according to the Material and Methods and amount of viral E1A copies was measured using real time PCR and presented as mean+/−SD. Cytotoxicity mediated by CRAd vectors at the glioma cells **(C)** and culture of adults non-malignant astrocytes **(D)**;* *p* < 0.05 vs CRAd-S-5/3, ** *p* < 0.05 vs CRAd-S-WT; *In vivo* cytopathic effect mediated by oncolytic vectors pseudotyped with fibers of adenoviruses belong to group B. **(E)** Therapeutic survival of mice in the presence of oncolytic vectors was measured using Kaplan-Meier survival plot. Mice received intracranial injection of U87 or U251 cells and 7 days later additional injection of one of the competent vector of AdWT, CRAd-S-WT, CRAd-S-5/3, CRAd-S-5/11, CRAd-S-5/35 or CRAd-S-pK7 vectors (100 IU/cell) were monitored twice per week over span of 50 days; **(F)** Efficacy of glioma inhibition mediated CRAd-S-pK7 and CRAd-S-5/3 vectors *in vivo*, *p* > 0.05.

To determine whether oncolytic vectors pseudotyped with adenovirus fiber B-subgroup could confer glioma cytotoxicity, we designed a panel of oncolytic vectors to test their anti-cancer effect using GBM cell lines and primary cells obtained from patients. First, we measured the ability of oncolytic vectors (Figure 2A) to transduce GBM cells, and to replicate specifically under the control of the human survivin promoter. As demonstrated (Figure 2B) A172, U118 and U87 glioma cells support replication of the CRAd-S-5/35 vector. In these cells, the magnitude of CRAd-S-5/35 replication was 10 fold (A172 (6.04 × 10(5)+/−3.5 × 10(5) and U87(1.1 × 10(5)+/−3.5 × 10(4)) and 1000 fold U118(2.24 × 10(6)+/−6.7 × 10(5) higher compared to the reference AdWT vector. On day 5 in U251 cells, CRAd-S-5/35 demonstrated lower replication (~6 fold) compared to AdWT.

To establish whether strong level of CRAd replication result in high GBM cytotoxicity, we performed a crystal violet test (Figure 2C). While, the CRAd-S-5/35 vector completely killed A172 glioma cells at 0.001 PFU/cell, the CRAd-S-5/11 and CRAd-S-5/3 recombinant viruses required 10 and 100 infectious units to kill the same number of cells. In both U251 and U87 cells, all vectors (CRAd-S-5/11, CRAd-S-5/35 and CRAd-S-5/3) exhibited the same level of glioma killing. In U118 cells, the CRAd-S-5/11 has 10–100 fold greater anti-glioma activity compared to CRAd-S-5/35, and CRAd-S-5/3 infections. Additionally, we observed that AdWT exhibits highly cytopathic effect on astrocytes at a dose of 0.001 PFU/cell (Figure 2D). For CRAd-S-5/11 and CRAd-S-5/35 vectors, we observed similar toxicity to that of AdWT-mediated one in normal human astrocytes. This observation highlights that in comparison to other fiber modifications (wt, 5/11 or 5/35) CRAd-S-5/3 vector can demonstrate elevated cytotoxicity at human glioma cells lines and exhibit lowest toxicity towards astrocytes.

In the next phase of the study we investigated whether the CRAd-S-5/3 vector could mount strong anti-GBM activity *in vivo* as well using mice xenografts developed after transplantation of U87 and U251 cell lines. As shown in Figure 2E, intracranial injection of CRAd-S-5/3 effectively suppressed U87 and U251 glioma tumor progression (55% survival at 43 days for U87 model, and 50% survival at 46 days for U251 model). While CRAd-S-5/3 vector showed some anti-glioma therapeutic efficacy detected by cell survival experiments *in vitro* and *in vivo*, AdWT, CRAd-S-WT, CRAd-S-5/11 and CRAd-S-5/35 failed to inhibit GBM progression and survibility significantly. Compared to the CRAd-S-pK7, we did not observe a significant difference in mice survival between single intracranial injection of CRAd-S-pK7, and CRAd-S-5/3 (NS, Figure 2F).

### CRAd-S-5/3 inhibits growth of patient derived primary GBM cells *in vitro*

**Figure 3:**
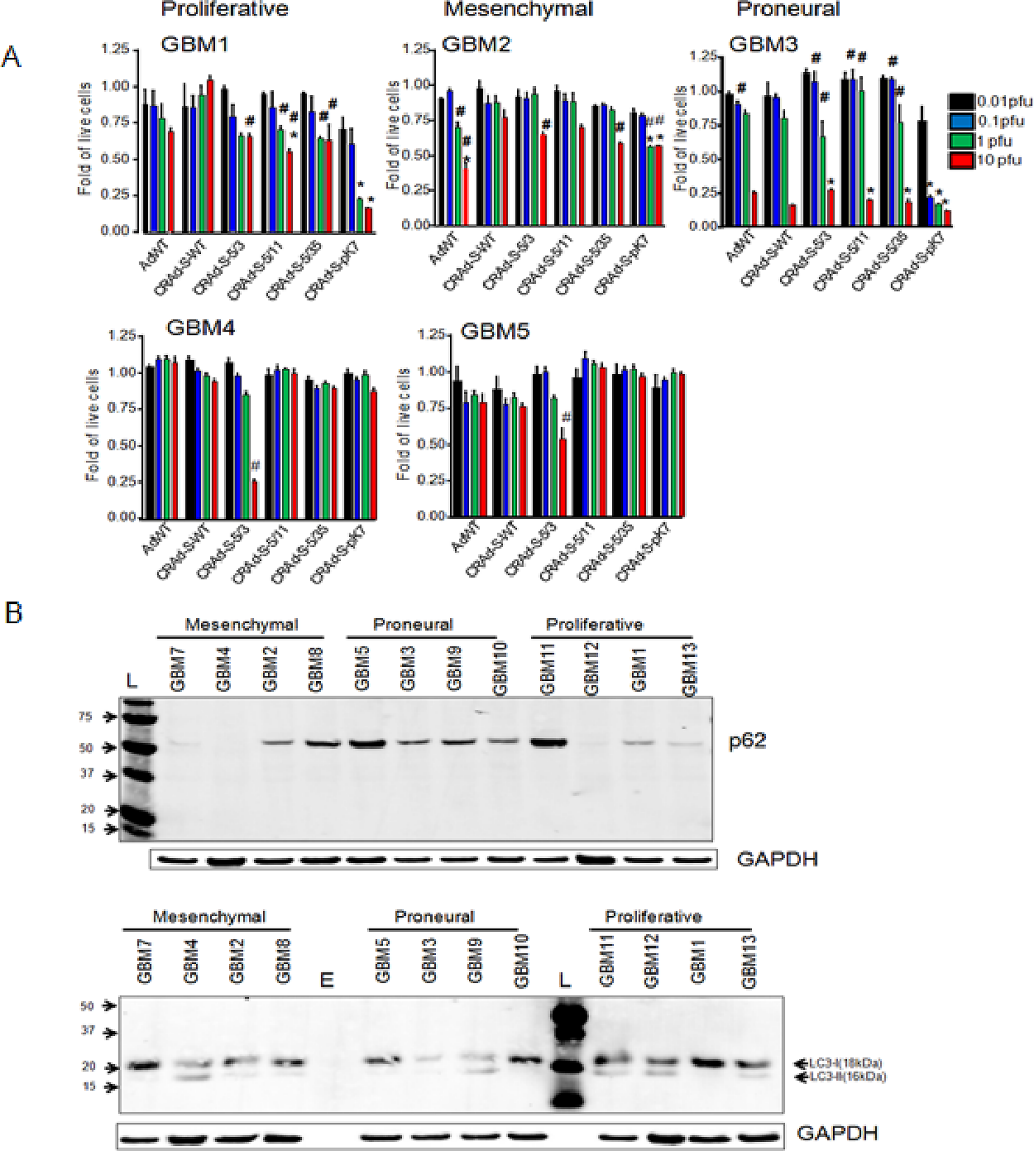
Patient derived gliomas cancer stem cells are resistant to the infection mediated by CRAds due to autophagy inhibition **(A)** Sensitivity of cancer cells (GBM1-GBM 5) to the infection of AdWT, CRAd-S-WT, CRAd-S-5/3, CRAd-S-5/11, CRAd-S-5/35 or CRAd-S-pK7 in various doses and at 5 days after virus infection was measured by MTT assay. Experiment was performed in quadruplicates; * *p* < 0.05 vs CRAd-S-5/3, # *p* < 0.05 vs CRAd-S-WT; **(B)** Expression of autophagy related proteins in the primary brain tumor cells. Cell lysates of primary glioma cells were subjected to immunoblot using antibodies recognizes autophagy related proteins such as p62 (part B, top) and LC3 (part B, bottom). GAPDH was used a loading control.

In general, there is growing concern that preclinical testing of oncolytic gene therapy using GBM cell lines may prove ineffective in clinics as primary tumor cells may lack cellular receptors required for adenoviral vectors to deliver the payload. Thus we investigated if survivin-dependent oncolytic vectors (Figure 2B and 2C) might prove equally effective against patient derived primary cells. Therefore, we infected patient derived GBM cells with CRAd vectors and determined their toxicity using trypan blue exclusion. In GBM2, 10 IU of CRAd-S-5/3 reduced proliferation of 70% GBM cells while the same vector reduced proliferation of 30% of GBM1 and GBM2 cells (Figure 3). Increased cytotoxicity of tumor cells in GBM2 isolated may be related to the fact that survivin promoter is active in these cells where, CRAd-S-5/3 required ~100 fold less viral particles to inhibit cancer cell proliferation. We also compared the activity of CRAd-S-5/3 versus CRAd-S-pK7 since the latter one is in the preclinal stage of development. We observed that in GBM1 and GBM3 cells, CRAd S-5/3 required 10–100 fold more viral particles to achieve similar cytotoxicity relative to CRAd-S-pK7 mediated infection. In other GBM isolates, such as GBM4 and GBM5, CRAd-S-5/3 demonstrated strong cytotoxicity (reveal the % of reduced proliferation) 40–73% of total cells were sensitive to CRAd-S-5/3 infection at 10 IU/cell. Data presented at Figure 3A also suggest that primary GBM cells restrict CRAd-S-5/3 cytotoxicity therefore increasing cytophatic effect mediated by CRAd is desired.

### Activation of autophagy improves anti-cancer effect of CRAd

Autophagy is a catalytic process which plays a dual function in cell survival and toxicity. In terms of adenoviral infection it was shown that autophagy negatively affects CRAd-mediated cytotoxicity. Specifically, autophagy is required to release viral progeny and effective CRAd infection [3, 7, 8, 27]. Our results suggest (Figure 3B) at GBM1 and GBM2 cells CRAd infection is influenced by inhibition of autophagy. For instance, we observed low levels of LC3-II pro-autophagy protein is correlated with moderate level of p62 which overall results in the low level of autophagy. In contrast, GBM12 and GBM3 cells, which exhibited strong autophagy that contributes to high levels of CRAd infection, highlighting elevated expression of cellular p62, moderate pro-autophagic LC3-II.

**Figure 4:**
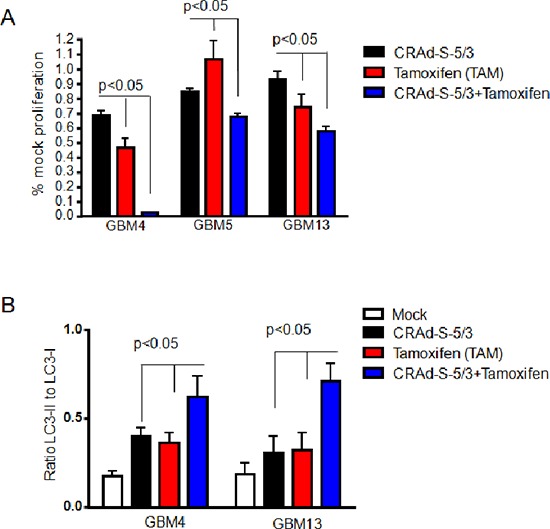
Effect of Tamoxifen on CRAd-S-5/3 oncolysis of CMV positive patient derived glioma stem cells **(A)** GBM4, GBM5 and GBM13 cells were infected with CRAd-S-5/3 (10 MOI per cell) for 24 hours or left untreated or treated with Tamoxifen (100nM, IC50). Sensitivity of glioma patient derived stem cells to the infection with CRAd-S-5/3 or combination treatment (CRAd-S-5/3+Tamoxifen) was analyzed for cell viability by MTT assay. MTT assay was performed at 5 replicates. Error bars for all MTT represent the mean plus/minus one standard deviation (Mean+/−SD). **(B)** Induction of autophagy mediated by Tamoxifen in the presence of CRAd-S-5/3 vector was assessed based on ratio of LC3-II and LC3-I expression detected by western blotting and measured via densitometry.

To improve anti-toxicity mediated by CRAd-S-5/3 vector we treated GBM4, GBM4 and GBM13 with Tamoxifen which is known to upregulate autophagy [28]. Data presented at Figure 4A suggest that Tamoxifen improves toxicity mediated by oncolityc CRAd-S-5/3 adenoviral vector (between 30–60% compared to CRAd-S-5/3 alone infection) via induction of autophagy which measured by upregulation of LC3-II expression in the presence of combination CRAd-S-5/3 and Tamoxifen (Figure 4B).

## DISCUSSION

Efficient transduction of target cells with adenoviral particles is a two-step process. First, attachment occurs when the viral fiber protein binds to the primary receptor. This event is followed by viral internalization via interactions on the cell membrane between penton bases and integrins α_V_β_3_, and α_V_β_5_. Despite the presence of primary receptors on the surface of many different types of cancer cells, some of glioma primary cells and cell lines [25] do not express CAR on their cell surface. Of note, CAR receptor is the primary receptor for human adenovirus type 5. As an alternative approach, we propose the targeting of the CD46 receptor which is present on the majority of glioma cells [25].

The field describing primary receptors for adenoviral type B is controversial. There were numerous attempts to identify the primary receptor for adenoviral type 3, including our own. All of these studies indicated out that adenovirus type 3 [29–31] along with adenoviral type 11 and 35 [32] recognize CD46 as the primary receptor. Most recently Wang H *et al* identified the primary receptor of adenovirus type 35 as desmoglein 2 (DSG2). Type 3 adenovirus uses an unknown receptor (X), while adenovirus type 11 uses receptor X and CD46 [13]. First in our study we measured expression of primary receptors (DSG2, CD46 and CAR) in glioma cells. The DSG2 molecule is universally expressed on the surface of not only cancer cells, but also non-tumor tissue (Figure 1B), and possibly on human astrocytes specifically when Ad5/35 and Ad5/11 application is decreased. Toivonen *et al*, and Hoffman *et al* demonstrated that enhanced transduction of glioma cells using the ad 5/35 modified vectors resulted in an increased anti-tumor response in head and neck squamous cell carcinoma (HNSCC), and glioma cell lines [33, 34]. Similar data were obtained in our study. Ad 5/11 and Ad 5/35 modified oncolytic vectors demonstrated significant cytotoxicity in glioma cell lines and primary specimens, which correlated with viral replication. Of note, due to low activity of survivin promoter compared to native E1 promoter, we observed some suppression of viral replication in CRAd-S-WT compared to AdWT virus. Targeting of glioma cells with survivin-driven CRAd improves upon retargeting of viral vectors to CD46 and/or DSG2 surface receptors as we detected in GBM3 primary glioma cells. Based on previous data [35], our evidence suggest that more virions are getting inside targets cells compared to WT modified vector. Thus, the efficacy of survivin driven B group modified oncolytic vectors was assessed *in vivo* using orthotropic U251 and U87 glioma intracranial xenografts models. We observed that there is no significant improvement in the glioma mice survival for Ad 5/11 and Ad 5/35 vectors. Moreover, Ad5/3 modification, which demonstrated some antiglioma activity, contributed to the CRAd-S-5/3 therapeutic efficacy. Of note, this efficacy was similar to CRAd-S-pK7, one of the candidates for a clinical trial. Earlier we compared the cytopathic effect of CRAd-S-5/3 vs CRAd-S-pK7 using glioma cell lines [36]. In our study, we demonstrated that CRAd-S-pK7 exhibited a cytopathic effect against proneural GBM3, and classical GBM1 glioma cells having high survivin promoter activity. For mesenchymal-type, we observed that all CRAds demonstrate similar therapeutic effects only at high dose (10 IU/cell), as cells became resistant to the low dose of CRAd infections. Of note, the mesenchymal subtype of glioma is characterized by more aggressive behavior and patients with mesenchymal subtype of glioma exhibit worse prognosis than proliferative, neural and proneural subtypes [37–39]. By comparing 5/3, 5/11 and 5/35 fiber modifications incorporated adenoviral capside, we demonstrate that 5/3 modification provided unique efficiency of glioma transduction *in vitro* and *in vivo* among tested oncolytic adenoviruses, as such in the future might lay foundation to be an efficient delivery system to the glioma.

Targeting of glioma cells with adenoviral vectors pseudotyped with fibers of group B1 could improve CRAd antiglioma cytopathic effect. Although, we observed significant glioma targeting with B pseudotyped modified vectors, incorporation of a polylysine motif (pK7) into fiber might demonstrate much stronger benefits for glioma virotherapy of glioma cells of proneural and proliferative subtype. Despite benefits of using CRAd for glioma experimental therapy, glioma cells provide various resistances to CRAd infection such as lack of primary receptor on the surface of glioma cells, low activity of promoter etc. Additionally, our analyses of patient derived glioma cells (Figure 1 and Figure 3 part B) suggest that patient derived glioma cells possess lack of autophagy. Since autophagy is required for CRAd progeny to be released from infected cells, new strategies to overcome glioma resistance to CRAd infection should be investigated. Although, this direction is out of scope for the current manuscript, our study laid a foundation for the future study by testing Tamoxifen [40] in combination with CRAd against patient derived glioma cells. Our data suggest that addition of Tamoxifen reduces proliferation of glioma cells and therefore contribute to cytopathic effect mediated by CRAd. Although, the precise mechanism of that contribution remains under investigation, out preliminary data suggest that Tamoxifen restores autophagy. One possible reason for reduced autophagy in primary GBM cells could be the fact that GBM cells express glycoproteins of CMV (Supplemental Figure 1A). Those proteins are likely can interact with autophagy cell machinery and suppress autophagy. Most recently, another scientific report suggests that the anti-viral drug Valganciclovir improves survival of GBM patients [41].

In conclusion, we found that CRAd-S-5/3 oncolytic adenoviral vector provide superior level of transduction of primary cells and glioma cells lines. *In vivo*, using U87 and U251 intracranial glioma models we determined that CRAd-S-pK7, a candidate for clinical trials, and CRAd-S-5/3 vectors exhibited similar efficacies of mice survival on glioma xenografts. Killing of primary GBM cells with CRAd-S-5/3 vectors faces challenges due to decreasing autophagy. Treatment of glioma cells with autophagy inducing agents such as Tamoxifen augments CRAd anti-glioma toxicity (Supplemental Figure 1B). These findings have a translational application since tamoxifen and oncolytic adenoviruses are both involved into clinical trials.

## MATERIALS AND METHODS

### Cell lines

The human glioma cell lines U87MG and U118 were purchased from ATCC. The U251 cell line was a kind gift from Dr. I Ivankov (Mayo Clinic, Rochester). The A172 and A549 cell lines were obtained from Dr. Z.B. Zhu (GTC, UAB). All cells were maintained in either DMEM/F12, or MEM media (both CellGro) supplemented with 10% fetal bovine serum (Hyclone, Loghan, UT). All patient derived glioma cells (GBM1-GBM13) were obtained from ongoing craniotomies performed at the Swedish Neuroscience Institute (Swedish Medical Center, Seattle, Wa) under written consent and were grown in media previously described [42]. All studies were approved by institutional Review Board of Swedish Medical Center. Cultures of human adult astrocytes (passage 3) were purchased from ScienCell (Carlsbad, Ca). OCT-frozen brain tumor specimens were collected and provided by Dr. A. Baryshnikov (RONC, Russia). At the time of procurement, primary brain tumor tissue were immediately frozen in optimum cutting temperature medium (Tissue-Tek) and stored at −80°C.

### Viral vectors

The replicative-competent CRAd-S-WT, CRAd-S-5/3, CRAd-S-5/11, CRAd-S-5/35 vectors have been created at RONC by homologous recombination between a shuttle plasmid containing the human survivin promoter upstream of the viral E1A gene and Ad5/5GFP, Ad5/3GFP, Ad5/11GFP and Ad5/35GFP plasmid backbones (these were a kind gift from A.Lieber, UW, Seattle, USA). The CRAd-S-pK7 and AdWT were previously described [43–45]and received from Dr. David Curiel (WashU, St.Lous) and from ATCC (Manassas, VA) relatively. Viruses were selected from single plaques on HEK293 cells [46], expanded in A549 cells, purified by double CsCl gradient ultracentrifugation, and then titrated using standard plaque assay on HEK293 cells [47].

### Subtyping of glioma cells

Total RNA is isolated from U251, U87, A172, U118 and GBM samples with Trizol (Invitrogen), and then cleaned with RNeasy MinElute Cleanup Kit (Qiagen). 1 ug of total RNA was used to generate 100 ul of cDNA using the High Capacity cDNA Reverse Transcription Kit (Applied Biosystems). Real-time PCR for 33 genes was performed on the ABI PRISM 7900 HT detection system using 33 taqman probes (Applied Biosystems) and taqman reagents under default conditions: 95°C for 10 minutes, 40 cycles of 95°C for 15 seconds, and 60°C for 1 minute with human beta-glucuronidase (hGUS) as endogenous control. All assays were done in triplicate. The expression level of each gene (Δct) for each sample is normalized to the hGUS expression level using the formula 2^−(Ct value of gene − Ct value of hGUS)^. For each gene we obtained the average (ct (μ) and standard deviation (σ). The standard scores (z) for all 33 genes per sample as follows: z_g_ = (Δct_g_ - μ/(σ_g_, where g (33 gene panel

Three components: 1. Mesenchymal (M), 2. Proliferative (P) and 3. Proneuronal (N) were calculated by taking the average of z scores of all genes belonging to the corresponding component.

$$\begin{array}{l} \text{M }=\mu \left({\text{z}}_{\text{g}}\right),\text{ where g}\in \text{component Mesenchymal}\\ \text{P }=\mu \left({\text{z}}_{\text{g}}\right),\text{ where g}\in \text{component Proliferative}\\ \text{N }=\mu \left({\text{z}}_{\text{g}}\right),\text{ where g}\in \text{component Proneuronal} \end{array}$$

Finally, the subtype is determined using the following reference range:

$$\begin{array}{l} \text{Mesenchymal }M>P+0.2,\;M>N+0.2\\ \text{Proliferative }P>M+0.2,\;P>N+0.2\\ \text{Proneuronal }N>M+0.2,\;N>P+0.2\\ \text{Prolifmes }P>N+0.2,\;M>N+0.2,\;\left|P-M\right|<0.2\\ \text{Mesneuronal }M>P+0.2,\;N>P+0.2,\;\left|N-M\right|<0.2\\ \text{Prolifneuronal }P\;>\;M+0.2,\;N>M+0.2,\;\left|N-P\right|<0.2 \end{array}$$

### Immunofluorescence of primary GBM specimens and cell cultures

For two-dimensional evaluation, 4-μm fresh-frozen sections were stained with immunofluorescent antibodies against desmoglein 2 (DSG2, Epitomics-Abcam, Burlingame, CA) in combination with DAPI (Invitrogen™, Life Technologies, Grand Island, NY). As a negative control, all stainings were performed without primary antibody. Fluorescently labeled species-specific secondary anti–immunoglobulin G (IgG) antibody (Invitrogen™, Life Technologies, Grand Island, NY) was applied for visualization of primary antibody. Images were captured with Olympus IX2-UCB DSU spinning disc confocal microscope (Olympus, Center Valley, PA) with an Evolve electron-multiplying charge-coupled device camera (Photometrics, Tucson, AZ). Dual-color immunofluorescence was performed at 600x magnification and a high–numerical aperture (NA, 1.2) oil-immersion objective. Images were acquired and stored in original SlideBook format (Intelligent Imaging Innovations Inc, Denver, CO). After image capture, files were saved in TIFF format.

### Rembrandt database patient samples analyses

Rembrandt gene expression data from malignant and non-malignant brain tumor patients was obtained from Rembrandt data portal (https://rembrandt.nci.nih.gov) using comprehensive Affymetrix U133 Plus 2.0 array that covers 47,000 cellular transcripts of whole genome.

### Viral toxicity test

Human glioma cell lines (U251, U87, U118 and A172), or patient derived cells were seeded at 3,000 cells/well in a 96 well plate, and allowed to grow for 24 hours at 37°C in DMEM/F12, or complete NSA medium (at 5 × 10^4 cells /well in 24-well plate for cells lines). The following day, cells were infected with AdWT, CRAd-S-WT, CRAd-S-5/3, CRAd-S-5/11 or CRAd-S-5/35 at 10, 1, 0.1, 0.01, 0.001 Infectious units (IU) per cell. After seven days, cells were stained with crystal violet, or MTT assays were performed.

### MTT-cell viability assay

Cell viability of glioma cells in the presence of various treatments was measured by Cell Proliferation Kit I (Roche Diagnostics Corp, Indianapolis, IN). Briefly, cancer cells were plated in 96-well plate (3,000 cells per well) for the next day treatment. On that day, cells were infected with various concentration of CRAd (10, 1, 0.1, 0.01 and 0.001 IU/cell) alone, or 24 hours later after additional with Tamoxifen at concentration (100 nM). After 1 hour of viral adsorption, virus containing media was replaced with media containing growth factors. At selected time points the MTT assay was conducted by adding 10 μl of MTT reagent and incubating for 4hrs. After, 100 μl of solubilzation solution was added to each well, and plates were incubated overnight before they were examined with the Microplate Reader (Model, Bio-Rad) at 570 nm with a reference of 655 nm.

Viral replication: Human glioma cells were seeded in DMEM/F12 medium (at 5 × 10^4 cells /well in 24-well plates and allowed to grow for 24 hours at 37°C. The following day, cells were infected with AdWT, CRAd-S-WT, CRAd-S-5/3, CRAd-S-5/11, or CRAd-S-5/35 at 1 Infectious unit (pfu) per cell. Cells and supernatant were collected and subjected to DNA isolation.

### Quantitative PCR

Total DNA was extracted from infected cells and supernatant using the DNeasy Tissue Kit (Qiagen, Hilden, Germany) according to the manufacturer's protocol. Gene expression was quantified by real-time quantitative PCR using SYBR Green PCR Master Mix (Applied Biosystems, Foster City, CA, USA) and primers recognizing the viral E1A gene [48]. DNA amplification was carried out using the Opticon 2 system (Bio-Rad, Foster City, CA, USA), and detection utilized the binding of the fluorescent dye, SYBR green. Each sample was run in triplicate. Results are presented as the average number of E1A copies per nanogram of DNA (E1A copies per ng DNA).

### Western blotting

Twenty micrograms of total protein was obtained from cells lines (U87, U118, A172 and U251), or patient-derived primary cell lines: mesenchymal (GBM2, GBM4, GBM6, GBM7, GBM8), proliferative (GBM3, GBM5, GBM10 and GBM11) and proneural (GBM1, GBM11, GBM12 and GBM13) subtypes. To measure the expression of human DSG2 (#5180, Epitomics-Abcam), CD46 (ab175096, Abcam, Cambridge, MA), Coxsackie adenoviral receptor (CAR, ab100811, Abcam, Cambridge, MA), survivin (2808, Cell Signaling, Danvers, MA), ATG5 (#2630, Cell Signaling, Danvers, MA), ATG7 (#2631, Cell Signaling, Danvers, MA), LC3 (#NB100–2331, Novus Biologicals, Littleton, CO), p62/SQSTM1 (ab109012, Abcam, Cambridge, MA), or cytomegalovirus based IE1 and CMVpp65 (Virusys, MD), membranes with immobilized proteins were incubated with the appropriate primary antibodies overnight. The following day, proteins were visualized using species-specific anti-IgG secondary antibodies (Li-Cor Biosciences, Lincoln, NE, USA). Images were acquired using Odyssey Imaging System (Model #1866, Li-COR Biosciences, Lincoln, NE).

### Orthotopic glioma model and CRAd treatment

Five to six week-old female mice were housed in a vivarium at N.N. Blokhin Russian Oncology Center. Entire protocol was reviewed and approved by the Animal Care Committee of Russian Oncology Center, RAMN. All procedures were conducted in accordance with the Guide for the Care and Use of Laboratory Animals (NIH). For orthotopic implantations of U87 and U251, a 2.5 μL RPMI-based solution that contained 25,000 (U87), or 50,000 (U251) cells was implanted into the right striatum of anesthetized mice. Briefly, mice were anesthetized with a mixture of ketamine/xylazine, and a 0.5 mm burr hole was then made through a scalp incision. The 30-gauge needle was left in place for 90 seconds, and then slowly withdrawn over a period of 60 seconds. After seven days, intracranial injection of AdWT, CRAd-S-WT, CRAd-S-5/3, CRAd-S-5/11, CRAd-S-5/35 or CRAd-S-pK7 vectors (100 IU/cell) was administered through the same hole used for tumor cell implantations. Mice were monitored bi-weekly and Kaplan-Meier survival curves were plotted.

### Statistical analyses

All T-tests were two-sided, and values were considered significant for *p*-value < 0.05. Kaplan-Meier survival curves were generated using Prism GraphPad v5.0, and log rank tests for survival experiments were conducted. One-way ANOVA, and Student's t-test were used to determine the significance between various groups.

## SUPPLEMENTARY FIGURE



## Abbreviations

GBM: (glioblastoma multiforme)
CRAd: conditional replicated vector
DSG2: desmoglein type 2
CD46: cluster of differentiation 46
