# Supplementary material for: Tamoxifen improves cytopathic effect of oncolytic adenovirus in primary glioblastoma cells mediated through autophagy
Source: Oncotarget. 2015 Mar 2;6(6):3977–87. doi: 10.18632/oncotarget.2897 (PMC4414167; doi:10.18632/oncotarget.2897)
Supplement: Supplementary file 1 [file oncotarget-06-3977-s001.pdf]

SUPPLEMENTARY FIGURE

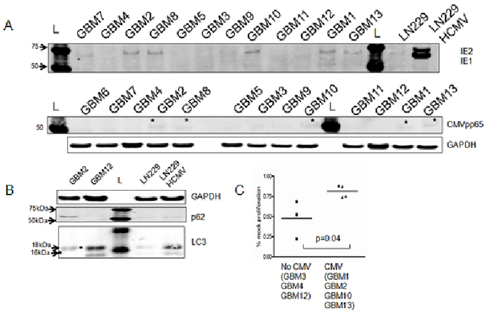

Supplementary Figure 1: Expression of CMV in the primary brain tumor cells correlates with autophagy inhibition. (A). Cell lysates of primary glioma cells were subjected to immunoblot using antibodies recognizes cytomegalovirus IE1(part A, top) and CMVpp65 (part A, bottom) proteins; **(B)** Average of CRAd-S-5/3 toxicity at dose of 10 MOI per cell at patient derived CSC expressing/lacking CMV proteins. **(C)** Average of CRAd-S-5/3 toxicity at dose of 10 MOI per cell at patient derived CSC expressing/lacking CMV proteins.
